# The views of mothers and GPs about postpartum care in Australian general practice

**DOI:** 10.1186/1471-2296-14-139

**Published:** 2013-09-25

**Authors:** Wendy Brodribb, Maria Zadoroznyj, Aimée Dane

**Affiliations:** 1 Discipline of General Practice, School of Medicine, The University of Queensland, Royal Brisbane and Women’s Hospital, Level 8, Health Sciences Building, Herston 4029, Australia; 2 Institute for Social Science Research, School of Social Science, The University of Queensland, 306 Michie Building, St Lucia 4072, Australia; 3 Queensland Centre for Mothers & Babies, School of Psychology, The University of Queensland, Hood St, St Lucia 4072, Australia

**Keywords:** Postpartum, General practice, Qualitative, Primary care, Australia

## Abstract

**Background:**

The postpartum period is a time of increased morbidity for mothers and infants under 12 months, yet is an under-researched area of primary care. Despite a relatively clear framework for involving general practitioners (GPs) in antenatal care, the structure of maternity service provision in some Australian jurisdictions has resulted in highly variable roles of general practice in routine postpartum care. This study aimed to investigate the views and experiences of mothers and GPs about postpartum care in general practice.

**Methods:**

This was a qualitative study of mothers and GPs in rural, regional and metropolitan areas of Queensland, Australia. Semi-structured interviews were conducted with 88 mothers and six general practitioners between September 2010 and February 2012. Interviews were recorded and transcribed verbatim. Data were analysed thematically and compared across groups.

**Results:**

Three main themes emerged: The relationship between the mother and GP; practice management; and GP visits. This paper focuses on the theme GP visits and its subthemes: recommendations for GP visits; scope of practice; and content of a routine visit. Recommendations about GP visits given to mothers varied by birthing sector, obstetric provider and model of maternity care resulting in confusion amongst mothers about the timing and role of GPs in routine postpartum care. Similarly, GPs voiced concerns about a lack of consistent guidelines for their involvement in routine postpartum care. Although ideally placed to provide primary care to mothers and their infants in the postpartum period, the lack of consistent guidelines for the role of GPs is of concern to both the GPs and early parenting women.

**Conclusion:**

General practice is an important source of postpartum care for mothers and provides a basis for ongoing support for the family. More consistent guidelines and better coordination with other care providers would benefit both mothers and GPs.

## Background

The postpartum period is a time of increased morbidity for mothers and infants under 12 months of age [1-5]. The World Health Organisation recommends mothers visit a health professional within two to three days of hospital discharge and then at four to six weeks [6]. Other guidelines recommend early infant review and assume a routine visit at six to eight weeks [7,8]. In Australia there are no consistent guidelines for routine postpartum visits, although many community services, including general practice, are available to women and infants following childbirth [9-11]. Consequently, postpartum services in primary care have been characterised as *‘inconsistent across jurisdictions, fragmented across disciplines and sectors, and currently do not adequately meet the needs of the population’*[12].

While Australia provides a universal public health system with coverage available to all citizens, it also has a private health insurance system that runs parallel and in competition with it [13,14]. Approximately 70% of Queensland births occur in the public sector [15] where women receive either midwifery or obstetric led care. The remaining 30% choose to birth in private hospitals under the care of a private obstetrician. Women who birth in public sector hospitals tend to be discharged around day two [15]. They are then eligible to be contacted by a registered midwife or child and family health nurse (phone or home visit) within 10 days of hospital discharge and are also encouraged to visit their general practitioner (GP) within this time frame [16]. Women who birth in private sector hospitals have longer hospital stays (approximately four days) [15] and return to their obstetrician around six weeks, often with no prior scheduled contact with a health professional. In a recent study 35% of women (16.6% from the private sector and 50.6% from the public sector) in Queensland visited their GP for the suggested mother and infant check within 10 days post-discharge [17]. In another study from regional Southern Queensland most mothers had visited a GP by three months postpartum [18].

Child and Family Health Centres, staffed by child and family health nurses, also provide support and advice as well as growth and development checks. Although they are freely available to all women and infants, in one area of Queensland attendance was as low as 50% in the first three months, [18] and there is a paucity of information about the attendance elsewhere.

In contrast to the information mothers receive about the timing of GP and health professional contact following birth, recommendations in the Personal Health Record book, given to all new parents for recording information about the infant’s birth, growth and development, include a GP visit within the first four weeks of life and then at two months. No comment is made regarding GP visits to review the mother’s health. These inconsistencies have the potential to confuse mothers and the health professionals who care for them about the most appropriate care during this period.

General practice is ideally placed to offer comprehensive, continuous and coordinated care to meet the needs of mothers and infants in the postpartum period and can be the foundation for extended family centred care [19]. While some research into postpartum care and general practice in Australia was conducted in the early 1990s, [20] little work has been undertaken since. This is despite significant changes in models of maternity care, in-hospital postpartum care, early discharge and availability of other community postpartum care providers. Importantly, only two qualitative studies have investigated how mothers view the role of general practice in postpartum care, [21,22] and one study investigated the role of GPs in well-child health care [23]. None of the studies have triangulated maternal and GP views or focused on the first two to three months postpartum. In addition, little work has been undertaken in other areas of the world.

The aim of this paper was to investigate postpartum care in general practice in Queensland from mothers’ and practitioners’ points of view and to identify issues that have an impact on effective service delivery.

## Method

Qualitative data presented in this paper were collected from two studies that recruited mothers and health professionals across a range of professions. However, as the role of different health professions varies, this paper only reports the methods and results of interviews with mothers and GPs.

The first small pilot study investigated the primary care services women accessed during the first eight weeks postpartum and was based in the capital city and one regional city in Queensland. Ten women in each site were recruited while in hospital post-birth and were contacted when their infant was approximately 10 weeks old. One GP in each of four practices in the regional city, known by one of the authors to have a high proportion of young families as patients, and six GPs in the capital city who were part of a maternity shared-care program (where antenatal care is shared between the hospital and the GP) at a tertiary hospital were sent letters inviting them to contact the researcher if they were interested in participating in the study. All were followed-up by telephone, fax or email three weeks after the letters were sent to ascertain their interest in being interviewed. Interviews for mothers and GPs were arranged for a mutually convenient time and place and were conducted between September 2010 and January 2012.

The second study, a state-wide evaluation of the Universal Postnatal Contact Service (a program including antenatal psychosocial screening, phone contact or a home visit within 10 days of hospital discharge and newborn and family drop-in centres in some areas), included a comparative case study of six sites in rural, regional and urban centres in Queensland. Researchers conducted face-to-face interviews with mothers and health professionals (including GPs) about postpartum care [16]. Mothers with children less than two years old were recruited through invitations at new mother’s groups, young parent’s groups and Child and Family Health Clinics, by advertising in print and social media and by word of mouth. Mothers were requested to contact the researchers if they were interested in participating and interviews were arranged for a mutually convenient time and place between August and December 2011. In one case study site, GP participation was sought through an advertisement in a newsletter for GPs in the region. GPs were also recruited through personal contacts and approached by mail to participate. They were then followed up via telephone after two weeks to ascertain interest in being interviewed. Interviews were conducted at the health professionals’ place of employment at a mutually convenient time during August and September 2011.

In both studies, after obtaining informed written consent from participants, interviews were recorded and transcribed verbatim. The interview outlines for mothers and community based health professionals for each study are found in Additional file 1. As the two studies asked very similar questions regarding postpartum care in the community, the data have been analysed together.

Each mother interview was read and data concerning general practice extracted. Content analysis was then undertaken using NVivo 9 software to identify emergent themes within and between interviews. There was regular discussion amongst the authors regarding the emerging themes to strengthen data interpretation. A similar process was undertaken for the GP interviews. A comparison of themes between the mother and GP interviews was then undertaken [24]. Investigator triangulation was used to ensure the reliability of thematic interpretations [25].

### Ethical approval

Ethics approval for both studies was obtained from the University of Queensland Behavioural & Social Sciences Ethical Review Committee. For Study 1 ethics approval was also obtained from the Queensland Health Human Research Ethics Committee and the Mater Health Services Human Research Ethics Committee.

## Results

In study 1, 18 mothers and four GPs were interviewed. Seventy mothers and two GPs were interviewed in Study 2. There was no response from the advertisement in the GP newsletter. The demographics for mothers and GPs for each study are available in Table 1.

**Table 1:** Demographic characteristics of participants in Study 1 and Study 2

	**Characteristics**	**Study 1**	**Study 2**
**Mothers**		**n = 18**	**n = 70**
Age	Mean (range) years	31.4 (21–41)	29.1 (21–39)
Parity	Primiparous	50%	52.8%
	Multiparous	50%	47.2%
Location	Metropolitan	50%	21.4%
	Regional/rural	50%	88.6%
Birthing sector	Public	50%	64.6%
	Private	50%	35.4%
Level of education	No university degree	66.6%	67.4%
	University degree	33.3%	32.6%
**General Practitioners**		**n = 4**	**n = 2**
Age	Mean (range) years	43.5 (35–55)	42.5 (38–47)
Gender	Male	2	0
	Female	2	2
Location	Metropolitan	2	0
	Regional/rural	2	2
Additional Qualifications	Fellow of the Royal Australian College of General Practitioners	2	2
	Fellow of the Australian College of Rural and Remote Medicine	0	1
	Royal Australian and New Zealand College of Obstetrician and Gynaecologists Diploma of Obstetrics	0	1
	Sexual Health Certificate	0	1
	International Board Certified Lactation Consultant	0	1

All GPs were parents and all conducted shared care for antenatal patients.

Following content analysis three main themes emerged– the relationship between the mother and GP, practice management, and GP visits. As large amounts of data were obtained, this paper only explores the theme GP visits and its three subthemes –recommendations for GP visits, scope of practice and content of a routine visit. Analyses of other data from these studies will be published in due course.

### Recommendations for GP visits

Women did not appear to be given consistent information about when they should visit their GP for a check-up for themselves or their infants following birth. The timing of the first postpartum visit in this cohort ranged from five days to two and a half months. Women from both the public and private sectors were most likely to visit the GP at six weeks for a ‘check’ or for the infant’s first immunisation at 6–8 weeks. For some women this visit was the first health professional contact they had had since hospital discharge.

There were considerable differences between women who birthed in the public and private sectors with regard to the information received and the timing of visits.

Women who birthed in the public sector were more likely to report having received explicit instructions to attend their GP in the first two weeks postpartum, especially if they were discharged within 48 hours of birth. This information was often given by a midwife attached to the maternity hospital who visited them at home after hospital discharge (domiciliary midwife).

…because the midwife was saying he needs a check-up from a doctor, so I took him and got him checked out. (Urban Mother(UM) age 27).

Other women followed recommendations from another health professional or information in the infant’s Personal Health Record book (a book given to mothers prior to discharge that suggests women attend their GP within the first four weeks). However, even women who participated in shared antenatal care found that the GP and hospital gave different information regarding postpartum review.

*She* [the doctor] *had different information from what the hospital told me… …The hospital said one thing and the doctor thought it was different… I think one thought we could do both her*[the baby] *check-up and mine at six weeks and the other thought four weeks for the baby and six weeks for me.(UM age 26).*

Many had little understanding of the reasons for visiting the GP after birth.

*I’m not sure* [why I needed to go], *but something to do with the baby, I needed to go. I think it was two weeks.(UM aged 28).*

Others tended to ignore advice to visit their GP, especially if they had ongoing visits from domiciliary midwives and no problems were evident, or they were multiparous.

Women who birthed in the private sector were given little information about ongoing care and were confused and uncertain if and when they needed routine follow-up after hospital discharge. For example, one mother who birthed in a large private hospital commented:

… we actually left the hospital a bit like, oh, so what are we meant to do now? Are we meant to go to the doctor or are we meant to have check-ups? We actually had no idea.(UM age 26).

While another primiparous mother from a regional centre reported:

*We were told that I didn’t need to see anyone or that I needed to take* [baby] *to see anyone, except at five weeks. (Rural Mother (RM) age 27).*

For many of these mothers there was a sense of abandonment by the hospital and their specialists, but they rarely considered visiting their GP.

*I really had a very hard time with him* [the baby] *so I was really kind of dumped, just left to my own devices.(RM age 27).*

One mother from the private sector felt that she would have been more confident and happier if it had been suggested to her to visit her GP within the first couple of weeks rather than waiting for five to six weeks.

If I knew to get her checked ....that it is strongly recommended that you have an appointment in the first 10 days or two weeks, even though it is hard to get out of the house… that would have been good to know I was on the right track. At least there is a light at the end of the tunnel.(RM aged 27).

Conversely, occasionally a mother was encouraged to see her GP for a check-up, rather than return to her specialist, even when the clinical situation was not straightforward. The mother of an infant born at 35 weeks and discharged with the infant, who was still jaundiced, one week later said:

I was told to see a GP within the next seven to 10 days to get him weighed and checked for his jaundice.(UM age 29).

With the divergence of recommendations about routine visits, it is not surprising that GPs lacked a consistent idea of when women should attend. While some GPs encouraged women and their infants to visit in the first five to ten days post-discharge and, if all was going well, again at six weeks for the mother and eight weeks for the infant, others recommended visits at one month, two months and then four months.

…neonatal check 7–10 days after delivery, especially in the public patient case, and then the next check, if everything is going to routine, at two months of age at the time of immunisation. (Urban general practitioner (UGP) 3).

GPs thought that the majority of women who received shared antenatal care returned in the first seven to 10 days postpartum. However, they were less certain that other women who birthed in the public sector, returned.

*I’d say about 60%… They’ve often been told that we’re busy; we haven’t got time* [and] *everything going fine. (Rural general practitioner (RGP 6).*

The GPs frequently did not see women who birthed privately until after six weeks postpartum, following their visit to the obstetrician and paediatrician. These women were usually not encouraged to visit their GP (by the hospital, specialist or GP) and GPs did not feel they had a role in the woman’s ongoing care.

What’s happening with these private patients … in that first six weeks …the majority aren’t coming to see me and that’s disappointing to me actually because I don’t believe it is the way the system should be working. I think the GP should be more heavily involved in that. (UGP 2).

Women who birthed privately or in a midwifery continuity of care program were seen to be disconnected from general practice care. Consequently, even though women may have had continuity of care (and/or carer) throughout their pregnancy, birth and postpartum period, they missed out on continuity of care/carer over the longer term.

…so they are separated from the general practice system until the point of immunisation and that’s where we seem to touch base with them again. (UGP3).

*The…push for continuity of intrapartum carer …has changed the dynamic again, and so it’s with some sadness that I see that, because I think we have a lot to offer, and I think continuity of care from my perspective extends well beyond 12 weeks* [gestation] *to six weeks* [postpartum]. *(UGP 6).*

This theme found a lack of clarity around the recommended timing and purpose of visits to the GP in the postpartum period leading to an inconsistent level of care for mothers and infants. GPs often felt sidelined during the postpartum period, especially for women birthing in the private sector or with a midwifery model of care.

### Scope of practice

Mothers’ views about the role of GPs in the care of infants differed, although there was little dispute about care for themselves. For some mothers the GP was the person they saw for any maternal or infant health issues and they had great confidence in their GPs ability.

I just go to my doctor every time.(UM age 35).

We…like our doctor, she was the one in charge… quite clearly the one in charge.(RM age 33).

For some mothers it was more convenient to attend their GP for routine weighs and checks rather than other sources of community postpartum support such as Child and Family Health Centres or pharmacy nurses. This may have been because of location, the services offered or the fact that their GP was also involved in their antenatal and intra-partum care and therefore had intimate knowledge of their circumstances.

I’ll get him weighed at the GP. I can just pop into my GP and they’ll weigh him for me.(UM age 25).

That way he’s got everything and knows what’s going on. (RM age 29).

For others, the GP was the person to go to if the infant appeared sick, or was not growing or developing normally, while other resources such as Child and Family Health Centres, telephone advice lines or mothers groups were used for infant behaviour problems and parenting advice. As well as conserving medical resources, this division of responsibility resulted from the view that GPs did not have the knowledge to manage day-to-day infant concerns. It also highlights the differing points of view about the scope of practice of medical care – is it illness focused or does it encompass comprehensive care of the patient and their family? One mother stated that:

My GP… isn’t there to tell me about suggestions on how to hold my baby when it’s got reflux and stuff. They’re there to fix an illness.(UM age 37).

While another thought:

… unsettled babies are something that doctors aren’t particularly good at and I don’t know if it’s terribly useful seeing …GPs about those sort of things to be honest because I think I know what they are going to say which is probably what my mothers’ group would say.(UM aged 35).

Even with immunisations there was a diversity of opinions with one mother stating that she would always go to the GP for immunisation:

[I go to the GP for vaccinations] *because … I was very concerned about side effects with vaccinations and I wanted to just have that more regular contact with the one person when it came to vaccinations.(RM age 33).*

While another mother always took her children to Child and Family Health Centre for immunisations because she did not think that it was something that had to be done at the GPs.

… *It’s a really good service to free up doctors. Like you don’t have to go to a doctor just to get your immunisations.(RM age 39).*

However, women were often informally referred to their GP by other community postpartum care providers if there were any issues about the mother’s or infant’s condition, or if the mother was particularly worried about a problem. Occasionally this referral was for investigations or treatment beyond the scope of practice of the other community postpartum care provider such as for prescription medications.

..they said, like, if we felt that he was becoming more jaundiced to go see our doctor and then request a blood test just to check his levels.(UM age 35).

I was a little concerned about his head shape…the health nurse I spoke to here said you should go and see your GP.(UM age 27).

Although she did not receive it, one mother thought that general practice was the ideal place for comprehensive care of mother and infant following her son’s diagnosis and treatment for pyloric stenosis.

*One thing I found with all the advice is there was not a holistic approach to* [baby’s] *health, my health and the breastfeeding. I could get advice for* [baby] *from* [the paediatrician], *I could get help from the GP about my mental health, but the GP didn’t offer any advice about breastfeeding. I saw the maternal and child health nurse about breastfeeding. … I would have been really appreciative to see a doctor who could have given me comprehensive advice on the whole problem rather than just part of the problem. It would have been helpful to have a bit more support as a mother trying to breastfeed a sick baby.(RM aged 28).*

The GPs interviewed did not see their role limited to routine checks and managing illness. Instead they thought their responsibility extended to assessing how the family was functioning and providing anticipatory guidance and education when appropriate. For example, one GP said:

You’ve got to be quite fluid....What is it that they’ve come to see you about? I’ve got to meet that need primarily. I’ve got to discharge my need which is keeping them safe and running through all the possible scenarios of something going terribly wrong… anything extra that I can plug in terms of education, that’s a bonus. (RGP7).

Therefore, while the GPs viewed their role as providing a broad range of care to mother and infant, the mother’s expectations of the role of the GP ranged from a person to go to if the infant was ill, to a person who should be able to provide or organise all-encompassing care for both.

### Content of a routine visit

When discussing the content of a routine postpartum visit, the interviewed GPs mentioned undertaking a thorough examination of the infant, enquiring about infant feeding and the mother’s physical and mental wellbeing.

…the baby is weighed, measured, neonatal examination, keep an eye on the mum and see what she’s looking like, how she’s managing, how she’s holding the baby and then go through all the things with mum, is she still bleeding? What type of feeding is she doing? How’s she managing? Is she sleeping? Any signs of depression? Briefly talk about intercourse, any problems with the labour, immunisations… Usually ask what the in-laws have said and remind them that both in-laws can be right about their baby or wrong about their baby but listen. (RGP6).

Appointment times ranged from 10 to 45–60 minutes with 30 minutes being common. The GPs acknowledged that the time allocated was often insufficient to manage problem with the mother or infant.

If you do pick up maternal issues, then that appointment can really blowout. (UGP4).

Mothers’ experiences of a postpartum/neonatal check varied. For some it was a comprehensive discussion and examination of the mother, infant, their social situation and how the family was managing with the new infant.

*She asked lots of questions like how I was going with breastfeeding, all about the labour and how we’ve gone since, how sore I was, how I’ve been healing…and then she did a lot of different tests* [on the infant]*.(RM age 21).*

Mothers found it particularly helpful when the GP asked specific questions or initiated discussion about problem areas – either physical or mental. These may have been problems that the mother was unaware of, thought were normal, or was too embarrassed to raise.

Then he looked at me and realised that I was iron deficient, so he realised that I needed a bit of tweaking too.(RM age 29).

However, this was not universal either with the breadth or depth of the consultation. In some circumstances the GP did not take the opportunity to look at the mother and infant as a unit, but only focused on the infant. This consultation and examination ranged from a general appraisal:to a detailed examination.

*He just looked at him and said “Yeah, he looks fine. We’re not going to weigh him or measure him or anything like that.”(UM age 30)*.

He weighed and measured her and answered a couple of questions, she has a hernia and something else that is not an issue now…listened to her heart and felt her tummy.(RM age 34).

This missed opportunity meant that some mothers did not seek a consultation for themselves as a follow-up to the birth or to assess any health problems. Some women were happy with the situation.

[What about a six week check?] *I have not bothered with that because I know I am fine.(RM age 37).*

Others would have done things differently in hindsight. One woman who did not see anyone for her own health in the first four months said:

*Looking back it is something that I probably should’ve done but at the time … I was more concerned about* [baby] *and looking after her.(RM age 33).*

From the information provided by the mothers in these studies there were inconsistencies in the scope and quality of GP postpartum visits. While some mothers and infants received a comprehensive discussion and examination, others underwent a very cursory examination with little discussion.

## Discussion

This study found, in keeping with other research, that mothers and GPs thought general practice was an important source of postpartum care [21-23,26-28]. However, in contrast to other countries [7] there was a lack of consistent and cohesive guidelines for community care of new mothers and their infants. This led to confusion around the timing of postpartum visits, GPs’ scope of practice, the content of the consultation and who was responsible for care. The two parallel health systems (public and private) in Australia [13] meant that no community follow-up after hospital discharge was recommended for one group of women until at least six weeks postpartum. GPs felt hesitant about their role in caring for women and infants whose antenatal care was conducted outside general practice as they were still ostensibly under the care of an obstetrician, paediatrician or hospital based staff, including those involved with a midwifery-led model of care. These women were less likely to be seen routinely postpartum in general practice with both the mother and GP being uncertain about the ‘ground rules’ for contact.

Very little research has been conducted on the most appropriate form of community postpartum care, particularly with regard to the role of general practitioners and other primary care physicians. In addition, many of the recommendations given are based on low grades of evidence. However, we know that women experience significant morbidity including tiredness, backache, urinary and bowel symptoms and breastfeeding difficulties, during the first few weeks postpartum [5,17]. Up to 20% also report problems with infant crying [29] and sleep disturbances [30]. Routine postpartum visits to a GP provide an opportunity to recognise and manage these challenges before they lead to unintended medium and long-term consequences for the mother and infant. Postpartum visits also provide a safety net for more serious conditions and a chance to offer anticipatory guidance about parenting, contraception and sexuality [31]. Because many women and their families already have an ongoing relationship with a GP, this postpartum visit allows reconnection of the mother and her infant with a person who will continue care in the long-term. For first-time mothers it can provide the impetus to engage with a GP who will provide extended family centred-care. There is a call for a more mother-centred approach to postpartum visits – with the timing depending on the individual needs of the mother [1]. However, the mother and health care providers must be given guidance about when to access assistance, and which problems require immediate attention.

GPs in this study thought that their scope of practice encompassed comprehensive and ongoing care for the mother and infant. A recent study also found that GPs thought they had an important role in providing well-child visits with an emphasis on health promotion and illness prevention. These well-child visits were often opportunistic with limited collaboration with other health professionals and little understanding of anticipatory guidance [23].

In contrast, mothers in this study had quite diverse views about the scope of practice of GPs; some thought that GPs’ main function was to manage illness, playing only a limited role in preventative health care and emotional support. Other studies have found that parents often have very clear views and expectations about the GP they choose for themselves and their infant [21]. In addition to managing any physical issues, women in these studies felt that primary care practitioners should proactively ask about their health and well-being, [22] assist with emotional issues [32] and provide a listening ear for maternal concerns [22].

While mothers in our study did not specifically mention rushed consultation times, they were selective about the reasons for visiting the GP, acknowledging that GPs were busy. The GPs admitted that postpartum consultations, especially if problems had to be addressed, often took longer than the booked appointment time. Short and rushed consultation times were raised repeatedly in other studies investigating postpartum care [19,21-23,32]. A lack of time was seen as the biggest disincentive for a mother to seek medical care for herself resulting in reluctance to divulge information, especially with regard to emotional issues [22,33]. Other issues such as out-of-pocket charges are also a deterrent for general practice postpartum care.

The ability to allocate sufficient time for routine postpartum visits is an important issue. As the population ages, and more time is allocated to managing the elderly with complex and chronic medical conditions, the time given for ‘non-acute and preventative care visits’ of necessity become shorter [34] and may become a threat to good quality postpartum care in general practice. GPs will have less exposure to infants and new mothers and may deskill, preferring to confine their practice to adults. Unless these factors are recognised and addressed, this important area of primary care may be neglected.

### Strengths and limitations

This is the first study to investigate the issue of postpartum care in general practice from both mothers’ and GPs’ points of view. We interviewed a large number of women from diverse localities and models of maternity care. However, we only interviewed a limited number of GPs who all had an interest in ante- and postpartum care. In addition, the mothers and GPs were not linked so we were unable to assess whether the experiences of the mothers matched the experiences of their GP. This study was based in Queensland, Australia whose postpartum care program may differ from other areas in Australia and overseas. Thus the views and ideas from the study participants may not be representative of GPs or mothers in other jurisdictions. Even so, this study points to the need for consistent guidelines/recommendations to prevent women receiving suboptimal care.

## Conclusions

General practitioners play an important role in postpartum care in the community but their potential to provide a broad range of care during this time period is often under-recognised, under-utilised and under-valued. This study has raised a number of important issues including the need for consistent recommendations and guidelines about postpartum primary care for all women in Australia. It also highlights the need to inform women and other service providers that GPs are available for postpartum consultations and provide a comprehensive service to mothers and their infants.

## Abbreviations

GP: General practitioner; UM: Urban mother; RM: Rural mother; UGP: Urban general practitioner; RGP: Rural general practitioner.

## Competing interests

The authors declare that they have no competing interests.

## Authors’ contributions

WB conceived the first study, participated in the design, data collection and analysis of both studies and drafted the manuscript. MZ participated in the design, data collection and analysis of the second study and reviewed the manuscript. AD participated in the organisation of the second study and in data collection and analysis. All authors read and approved the final manuscript.

## Authors’ information

WB is a general practitioner and academic with a specific interest in postpartum care in the community. MZ is a social science researcher with experience in health service delivery to mothers and their infants. AD is a research assistant with the Queensland Centre for Mothers & Babies.

## Pre-publication history

The pre-publication history for this paper can be accessed here:

http://www.biomedcentral.com/1471-2296/14/139/prepub

## Supplementary Material

Additional file 1 **Interview outlines.** Outline of interviews for Study 1 and Study 2. Click here for file
