# Supplementary material for: The views of mothers and GPs about postpartum care in Australian general practice
Source: BMC Fam Pract. 2013 Sep 25;14:139. doi: 10.1186/1471-2296-14-139 (PMC3851599; doi:10.1186/1471-2296-14-139)
Supplement: Additional file 1 — Interview outlines. Outline of interviews for Study 1 and Study 2. [file 1471-2296-14-139-S1.pdf]

Additional file 1 – Interview outlines for mothers and health professionals

Study 1

Mothers

- Participant demographics (age, parity, public/private, type of birth)
- Medical and breastfeeding problems – maternal or infant - at hospital discharge and subsequently
- Mother and baby services used in the first months postpartum
- How she found out about the service – including referral from another service provider or maternity service
- What advice she was given – particularly related to infant feeding
- Whether the service was helpful
- What would have improved the help and support she received in that period.

Health professionals

- Participant demographics (age, qualifications, work experience)
- What services do they provide for women in the first month postpartum
- How do women access the service? – referral methods and advertising of the service
- The typical age of the baby when first seen at the service
- Typical problems seen and advice given
- Typical length of consultation
- Provision for ongoing follow-up and recommendations for follow-up or routine assessment
- Consultation and collaboration with and referral to other service providers
- Suggestions for improvements in service provision to mothers and their babies

Study 2

Mothers

- Participant demographics (age, parity, public/private, type of birth)
- Duration of hospital stay, and evaluation of it
- Hospital to home discharge experience - Its type, character, and perceptions of the experience
- Quality of post-birth experience following discharge home - assessments of its quality, availability, ease of access, and usefulness.
- Postnatal health contact: patterns, perceptions, evaluation

- Mother’s health, wellbeing, social and emotional support
- Baby’s health, feeding and wellbeing
- What could be done better / what *i*mprovements could be made to help new mums and their babies?

Health professionals

- Participant demographics (age, qualifications, work experience)
- What postnatal services they offer to women?
- How are women informed about the available services?
- Who accesses their service?
- How old are babies when they first come to the service and what is the reason for attendance?
- What happens at visits to their service?
- How is continuity and integration of maternity care between hospital and community managed?
- What provision is there for ongoing follow-up and recommendations for routine assessment?
- What methods of consultation, collaboration and referral are there with other service providers?
- What is their perception of the UPNCI?
- What is their perception of the effect it has had on mothers?
- What improvements could they suggest to postnatal services?
